# Mechanisms and prognostic impact of myocardial ischaemia in hypertrophic cardiomyopathy

**DOI:** 10.1007/s10554-023-02894-y

**Published:** 2023-06-26

**Authors:** James A. Coleman, Zakariye Ashkir, Betty Raman, Alfonso Bueno-Orovio

**Affiliations:** 1 Department of Computer Science, University of Oxford, Oxford, UK; 2 Oxford Centre for Clinical Magnetic Resonance Research, John Radcliffe Hospital, University of Oxford, Oxford, UK

**Keywords:** Ischaemia, Hypertrophic cardiomyopathy, Arrhythmic risk, Sudden cardiac death, Fibrosis, Perfusion CMR

## Abstract

Despite the progress made in risk stratification, sudden cardiac death and heart failure remain dreaded complications for hypertrophic cardiomyopathy (HCM) patients. Myocardial ischaemia is widely acknowledged as a contributor to cardiovascular events, but the assessment of ischaemia is not yet included in HCM clinical guidelines. This review aims to evaluate the HCM-specific pro-ischaemic mechanisms and the potential prognostic value of imaging for myocardial ischaemia in HCM. A literature review was performed using PubMed to identify studies with non-invasive imaging of ischaemia (cardiovascular magnetic resonance, echocardiography, and nuclear imaging) in HCM, prioritising studies published after the last major review in 2009. Other studies, including invasive ischaemia assessment and post-mortem histology, were also considered for mechanistic or prognostic relevance. Pro-ischaemic mechanisms in HCM reviewed included the effects of sarcomeric mutations, microvascular remodelling, hypertrophy, extravascular compressive forces and left ventricular outflow tract obstruction. The relationship between ischaemia and fibrosis was re-appraised by considering segment-wise analyses in multimodal imaging studies. The prognostic significance of myocardial ischaemia in HCM was evaluated using longitudinal studies with composite endpoints, and reports of ischaemia-arrhythmia associations were further considered. The high prevalence of ischaemia in HCM is explained by several micro- and macrostructural pathological features, alongside mutation-associated energetic impairment. Ischaemia on imaging identifies a subgroup of HCM patients at higher risk of adverse cardiovascular outcomes. Ischaemic HCM phenotypes are a high-risk subgroup associated with more advanced left ventricular remodelling, but further studies are required to evaluate the independent prognostic value of non-invasive imaging for ischaemia.

## Background

Hypertrophic cardiomyopathy (HCM) is the most common inherited heart disease (1:200–1:500 [1]), a leading cause of sudden cardiac death (SCD) in the young, and a common cause of heart failure and atrial fibrillation in adults [2]. The clinical course of HCM is complicated by multiple factors that can interact and exacerbate the phenotype, including left ventricular outflow tract (LVOT) obstruction, mitral regurgitation, diastolic dysfunction, arrhythmias, autonomic dysfunction and myocardial ischaemia [2]. Myocardial ischaemia has been identified as an area of investigative importance [3], because of its association with adverse left ventricular (LV) remodelling and poor clinical outcomes in early studies of HCM [4] and other cardiovascular diseases.

Despite ischaemia being considered a significant contributor to the natural history of HCM [3], recommendations to assess ischaemic burden are absent from clinical guidelines, and HCM-specific strategies to mitigate ischaemia remain limited. This is in part because the treatment of ischaemia in HCM is complicated by multiple pathophysiological mechanisms, with many patients demonstrating evidence of myocardial infarction in the absence of epicardial coronary stenoses [5–8], such that multiple other pro-ischaemic mechanisms must be considered alongside therapeutic strategies other than revascularisation [6]. Despite their importance, the HCM-specific pro-ischaemic mechanisms are not yet fully understood.

Furthermore, as the major cause of SCDs in the general population, the assessment of ischaemia in HCM may address some limitations of SCD risk stratification, which has suboptimal sensitivity [9]. This may be particularly true in young HCM patients [10], who carry significant SCD burden, and in whom acute myocardial infarction is possible [11], because a structural substrate for lethal arrhythmias is frequently absent in juvenile SCDs [12]. Although ischaemia is hypothesised to contribute to SCD events, the prognostic value of imaging for ischaemia in HCM is not yet well established.

This review therefore aims to (1) reflect on the prevalence of ischaemia in HCM and its multifactorial causes, to establish potential therapeutic targets for the treatment of ischaemia without epicardial coronary stenoses; and (2) assess the prognostic impact of imaging for ischaemia in HCM in relation to other markers of disease severity (hypertrophy and fibrosis), to evaluate the role of ischaemia on imaging as a potentially novel SCD risk factor.

To address these aims, the present study first reviews the frequency of myocardial ischaemia in HCM on non-invasive assessment, with a focus on modern advancements made in cardiovascular magnetic resonance (CMR) imaging. The pathophysiological mechanisms underlying the development of ischaemia in HCM are then explored, and how these may contribute to adverse LV remodelling and symptomatic status. The role of imaging for myocardial ischaemia in HCM as a risk marker for adverse outcomes including arrhythmias and heart failure is further considered, through review of follow-up studies. Finally, the role of myocardial ischaemia is discussed as a therapeutic target, through reviewing the latest clinical trials targeting metabolic and vascular dysfunction in HCM. We expect these findings to contribute to the scientific understanding and clinical management of myocardial ischaemia in this high-risk group of patients.

## Imaging of myocardial ischaemia in HCM

**Fig. 1:**
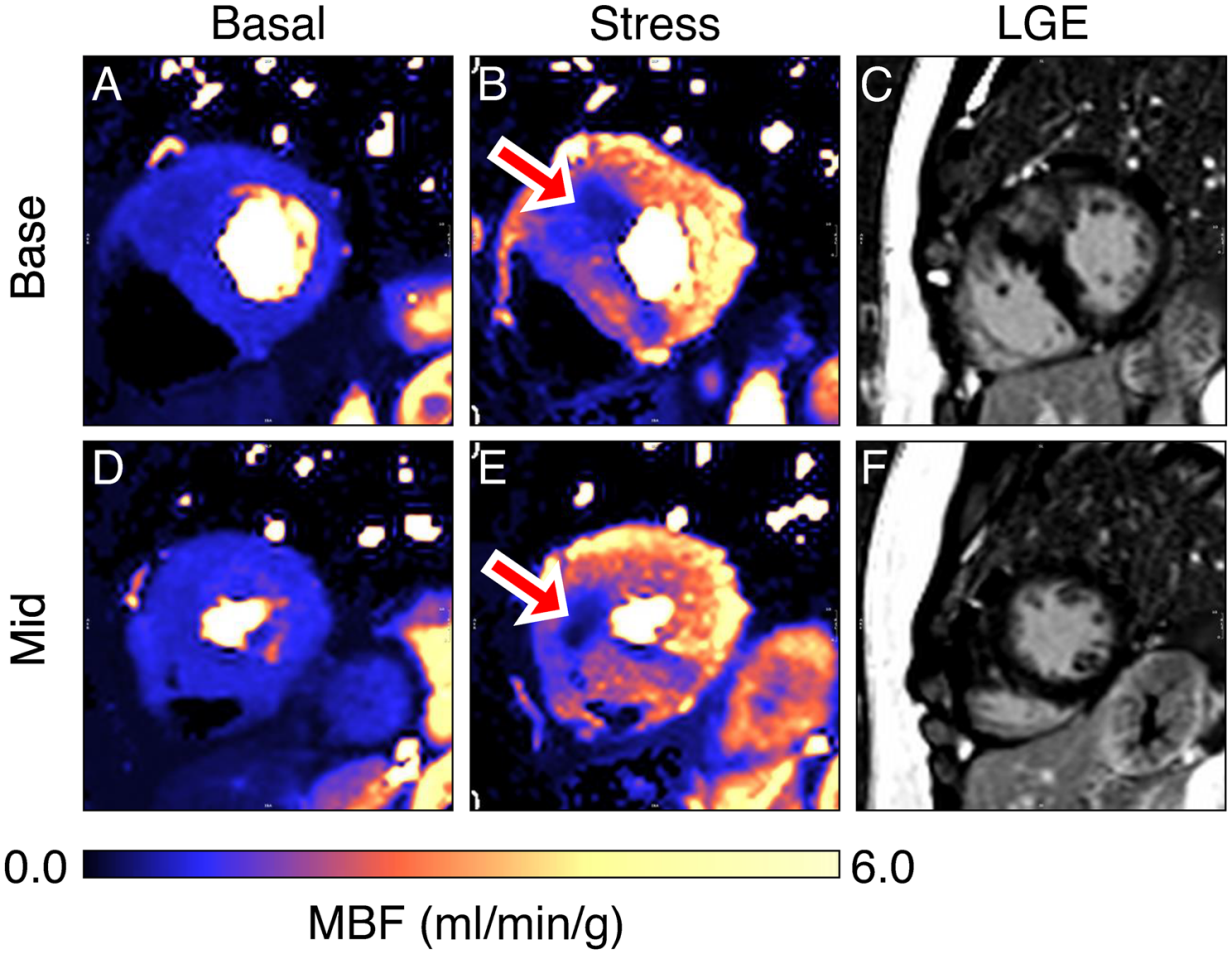
Stress perfusion defects in HCM on perfusion CMR. (A, D) Basal and (B, E) adenosine-stress MBF on perfusion CMR in the (A, B) base and (D, E) mid slices of a 40-year-old woman with sarcomere mutation positive HCM, showing stress perfusion impairment (denoted with arrows) in the maximally hypertrophied anterior wall and septum. (C, F) Late gadolinium enhancement in the same patient for the base and mid slices, respectively, showing dense focal enhancement in the hypertrophied anteroseptum

Perfusion measurements are commonly used as a surrogate of ischaemia, as integrated measures of flow through both the epicardial coronary arteries and the microcirculation. Regional perfusion defects are characteristic of the HCM phenotype and present as regions of impaired myocardial blood flow (MBF) at rest or during exercise/pharmacologically induced hyperaemia (Fig. 1). Perfusion impairment is commonly inferred from a reduced ratio of hyperaemic MBF to rest MBF, termed myocardial perfusion reserve, and is generally considered an acceptable surrogate for ischaemia [3].

Table 1 summarises the non-invasive imaging studies that have assessed the presence of perfusion defects in HCM patients, through CMR, echocardiography and nuclear imaging. However, not all imaging modalities are equally accurate. Perfusion defects on Th-201 scintigraphy, for example, correlate poorly with acidosis [13].

**Table 1:** Studies reporting prevalence of perfusion defects among HCM patients

References	Year	Imaging modality	N/n	Summary of findings
[14]	1987	Th-201 emission CT	72/0	57% any, 24% fixed, 33% reversible
[15]	1989	Scintigraphy	28/0	39% any
[16]	1989	Th-201 emission CT	29/0	3% fixed, 52% stress
[17]	1993	Th-201 emission CT	82/0	33% fixed, 39% reversible
[18]	1993	Scintigraphy	23/0	78% any
[19]	1996	Scintigraphy	17/0	71% any
[20]	1996	Scintigraphy	94/0	20% fixed, 21% reversible
[21]	1998	Scintigraphy	105/0	69% any, 30% fixed, 60% reversible
[22]	1998	Th-201 emission CT	216/0	40% any, 25% fixed, 22% reversible
[23]	2004	99mTc SPECT	101/0	54% any, 28% fixed, 41% reversible
[24]	2008	CMR	37/0	46% rest
[25]	2008	Echo	68/74	46% CFR < 2
[26]	2010	SPECT + MCE	33/23	100% any (MCE), 0% any (SPECT)
[27]	2012	PET	33/0	72% reversible
[28]	2013	PET	47/0	72% any
[29]	2013	CMR	86/0	57% any
[30]	2014	CMR	33/0	87% any
[31]	2014	CMR	35/0	31% MPR < 1
[32]	2014	CMR + Echo	148/0	7% any
[33]	2015	CMR	12/9	18% rest, 73% stress
[34]	2015	CMR	80/0	30% rest
[35]	2016	Echo	706/0	38% CFVR < = 2
[36]	2016	CMR	30/0	60% stress
[37]	2018	CMR	13/0	0% rest, 54% stress
[38]	2019	CMR	101/30	79% any
[39]	2019	CMR	35/0	71% MPRI < 1.4
[40]	2020	CMR	115/0	42% stress
[41]	2020	CMR	105/0	45% MPRI < = 2
[42]	2021	CMR	449/0	84% stress
[43]	2021	CMR	50/28	20% any
[10]	2021	99mTc SPECT	91/0	77% any, 24% fixed, 53% reversible
[44]	2021	CMR	75/0	91% any

Perfusion defects were mostly identified by visual assessment, and in some cases assessed quantitatively with perfusion reserve/flow measurements [25, 31, 35, 39, 41]. Studies using visual assessment identified patients with perfusion defects occurring at rest, stress, exclusively stress (reversible), and both at rest and stress (fixed)

*CMR* cardiovascular magnetic resonance, *MCE* myocardial contrast echocardiography, *PET* positron emission tomography, *SPECT* single-photon emission computed tomography, *CT* computed tomography, *CFR* coronary flow reserve, *MPR* myocardial perfusion reserve, *CFVR* coronary flow velocity reserve, *MPRI* myocardial perfusion reserve index, *N* number of HCM patients, *n* number of non-HCM controls

Since the last review of imaging techniques for myocardial ischaemia in HCM in 2009 [3], ischaemia assessment with CMR has been widely adopted. Perfusion CMR boasts MBF quantification at high resolution, without radiation exposure, and has proven high sensitivity for coronary artery disease diagnosis in the general population [45]. The capability of CMR to measure multiple modalities further enables the assessment of ischaemic and fibrotic burden in quick succession (see later Table 2). Moreover, as ischaemia results from an imbalance between oxygen supply and demand, modalities such as blood oxygen level dependent (BOLD) CMR that is sensitive to myocardial oxygenation can provide further insight to oxygen supply and demand in HCM [46].

Across the perfusion imaging studies in Table 1, ischaemia is frequently identified in HCM patients, with the largest perfusion CMR study to date identifying inducible perfusion defects in 84% of their cohort [42]. Because perfusion defects were typically identified on visual assessment, often the severity of impairment was omitted. However, quantitative assessments have identified a subset of 21–31% of patients in whom regional perfusion can fall during vasodilator stress [31, 38], a finding suggestive of severe microvascular dysfunction.

## Pathophysiological mechanisms of ischaemia in HCM

With perfusion defects often remote from coronary territories [37], and in young patients likely without coronary stenoses [10], myocardial ischaemia in HCM is multifactorial in origin. Numerous post-mortem, biopsy and imaging studies have investigated the micro-, macrostructural and metabolic mechanisms underlying ischaemia in HCM.

### Microstructural abnormalities in HCM

#### Small vessel disease

Structural abnormalities in the small blood vessels that supply the myocardium are a common finding in the histology of HCM hearts [47, 48]. As many as 56–83% of HCM patients have small vessel disease to some degree [49–52]. The abnormalities typically include marked thickening of the vessel walls, luminal narrowing of small intramural coronary arteries [53] and increased arterial stiffness [54]. The luminal area of arterioles, as a percentage of total vascular area, has been measured to be 13–30% lower in HCM patients than in controls [55–57], with 14% of small vessels in one study having an external diameter:lumen ratio ≥ 3 (normally < 2.5) [58]. Other studies found small vessel disease in 92% of myocardial specimens taken from 57 HCM patients [59], and that the HCM myocardium had 30 × more abnormal intramural coronary arteries per section on average than controls [51]. The degree of small vessel disease may be particularly severe in patients with heart failure [12].

#### Reduced density of small vessels

Numerous studies have measured the density of small vessels in hypertrophied HCM septal/LV tissue samples and have found this to be 21–44% lower than in control patients [54, 56, 57, 60–63]. With no convincing association with genotype [61], reductions in small vessel density may be linked to hypertrophy in HCM and are more severe in end-stage disease patients, such as those undergoing heart transplantation, than in patients referred for myectomy [62]. The reported association between reduced small vessel density and perfusion reserve blunting suggests that this is another cause of ischaemia [57].

### Macrostructural forces in HCM

#### Left ventricular outflow tract obstruction

LVOT obstruction is a hallmark feature of HCM and is defined as a peak LVOT pressure gradient ≥ 30 mmHg. LVOT obstruction may be present at rest (in up to 51% of HCM patients [41, 64–66]) or develop during exercise (in 33–62% [65, 66]). Severe obstruction (≥ 50 mmHg) may be seen in up to 20% of patients [35]. Even in the absence of hypertrophy, LVOT obstruction may be present due to systolic motion of the anterior mitral valve leaflet towards the LVOT [67]. This typically results from lengthening of the anterior mitral valve leaflet, abnormal chordal-mitral valve attachment or bifid papillary muscle hypermobility [67].

LVOT obstruction is associated with reduced perfusion reserve [25, 35, 41, 68] particularly in the left anterior descending artery which supplies the anterior wall and septum [68, 69], reduced hyperaemic MBF [70], reduced endo-epicardial hyperaemic MBF ratio [71], reduced perfusion upslope (a measure of contrast agent wash-in time) [72] and an additional hemodynamic forward deceleration wave in systole [73]. These associations may be explained by coronary hypoperfusion and increased oxygen demand, as greater myocardial work is required to overcome the obstruction.

Despite these reported associations, other studies have found no relationship between LVOT obstruction and ischaemia, and infarction has been reported in non-obstructive HCM [11], confirming the role of other mechanisms including microvascular dysfunction [4, 10, 27, 32, 44].

#### Effects during diastole and systole

Diastolic dysfunction, partially related to sarcomeric mutations, is an early feature of HCM in many patients [74, 75]. With myocardial blood flow greatest during diastole, constrained diastole results in impaired perfusion [76]. In a study by Raphael et al., invasive measurements of coronary pressure and flow showed that diastolic dysfunction in HCM led to impaired decompression of the microcirculation [73], and these effects could be exacerbated by exercise [77].

During systole, hypertrophy causes excessive compression of intramyocardial blood vessels, leading to abnormal coronary haemodynamic forces [73], which can manifest as systolic flow reversal in septal perforator arteries of HCM patients [69, 78–80] even in the absence of LVOT obstruction [73].

#### Myocardial bridging

Another potential mechanism of perfusion impairment among HCM patients is myocardial bridging, which is when a segment of a coronary artery tunnels through the myocardium rather than over it. This is seen in 23–41% of HCM patients compared to 5–7% of the general population [81, 82]. Multiple case reports highlight bridging as a possible cause of ischaemia [83–86], however surgical correction of myocardial bridging remains controversial [87, 88].

### Genetic and metabolic factors

In multiple studies of genotyped HCM patients, vasodilator stress BOLD CMR has detected impaired myocardial oxygenation even in pre-hypertrophic carriers of sarcomeric mutations, despite preserved perfusion [46, 89, 90]. One possible explanation for this dissociation in oxygenation and perfusion often observed in the early phase of the disease is that myocardial oxygen demand may be increased even in the absence of hypertrophy [91–94]. In line with this, experimental non-hypertrophic murine models of sarcomeric mutations have demonstrated increased oxygen expenditure arising from energetic inefficiency associated with sarcomeric mutations [95]. In HCM patients with overt hypertrophy, the degree of stress oxygenation may be as severe as that seen in severe aortic stenosis [90, 96].

## Relationships between markers of disease severity and ischaemia in HCM

### Myocardial hypertrophy and ischaemia

The greater the degree of hypertrophy (quantified as wall thickness or LV mass), the greater the degree of perfusion impairment (typically quantified as rest or hyperaemic MBF, perfusion reserve, flow velocity, or as the presence of visual defects). This has been reported both at a segmental level [26, 29, 31, 33, 34, 38, 41, 46, 48, 70, 72, 97–101] and in HCM patients on average [7, 15, 22, 27, 40, 44, 68, 70, 71, 78, 79, 102]. In addition to reductions in capillary density [61], hypertrophy was frequently associated with enhanced luminal narrowing of small vessels [50, 55, 56, 58].

A further macroscopic explanation is that the increase in muscle mass characteristic of HCM is inadequately supplied by the major coronary arteries, which have reduced luminal volume per unit myocardial mass [103–105]. Heterogeneous flow among major coronary arteries secondary to variable regional demand is a further consequence of hypertrophy [48, 68, 69, 105].

Although perfusion defects are overall more prevalent in cohorts with hypertrophy (Table 1), 20% of HCM mutation carriers without hypertrophy may still have perfusion defects [43], and even in hypertrophic cohorts, 30–40% of patients may have perfusion defects in segments with only mild hypertrophy [15, 106]. When compared to controls, even non-hypertrophied segments in HCM have reduced perfusion reserve on average [38].

### Segmental and transmural distribution of myocardial ischaemia

Two studies reported perfusion defects [30] and exercise wall motion abnormalities (WMAs) [32] to be most frequently septal, which is consistent with the septum and anterior LV wall being the most frequently hypertrophied regions in HCM [64]. In another cohort, the septum and inferior segments were most affected by perfusion defects [101]. Perfusion defects have also been reported as primarily located in the septum in prehypertrophic HCM mutation carriers [43], which may be explained by small vessel disease reported as mostly affecting the septum in histological analyses [51, 59]. However, Villa et al. reported a more diffuse burden of hypoperfusion in a cohort without severe hypertrophy [36], consistent with the widespread distribution of small vessel disease in a different histological analysis by Varnava et al. [58].

There are also conflicting reports on the segmental distribution of perfusion defects in SPECT imaging studies [22, 23, 80, 107], which may be due to partial volume effects, given the wall thickness-dependent sensitivity of SPECT imaging. Indeed, PET studies, which have similar problems with partial volume effects due to its low resolution, sometimes report similar impairment in MBF between the septum and LV free wall [4, 70, 108], with two exceptions [28, 109].

Perfusion impairment in HCM is predominantly subendocardial [29, 31, 38, 42, 43, 70, 71, 97, 101, 106, 108], although transmural hypoperfusion has also been reported [38, 98].

### Myocardial fibrosis and ischaemia

Repeated episodes of ischaemia have been implicated in fibrosis accumulation and extensive scarring in HCM [47, 53]. A longitudinal study of HCM patients with combined late gadolinium enhancement (LGE) to assess fibrosis burden and stress perfusion imaging found that patients with impaired perfusion reserve had a greater increase in LGE mass over time [39]. Indeed, multiple histopathological studies have shown that the presence of diseased small intramural coronary arteries and reductions in microvascular density are topographically correlated with the presence of fibrosis [12, 47, 51, 52, 59, 110], with one exception [58]. This was similarly reported among patients [55], with Kwon et al. reporting that the presence of small vessel disease is independently associated with 14 × increased risk of myocardial scarring [50]. The association with replacement fibrosis appears primarily in end-stage HCM [59].

However, whereas ischaemia is predominantly subendocardial in HCM [29, 31, 38, 42, 43, 70, 71, 97, 106, 108], fibrosis is predominantly mid-wall in HCM [33, 53, 99, 111–116]. This transmural dissociation has been reported directly on imaging [29, 33]. Segment-wise associations between fibrosis and ischaemia have also been reported on imaging in HCM (Table 2), however it remains to be seen whether this is due to the confounding effect of disease severity reflected by wall thickness [31], or due to difficulties in discerning regions of ischaemia and fibrosis [36].

**Table 2:** Imaging studies in which segment-wise associations between ischaemia and fibrosis have been analysed

References Modality	N/n	Epi-endo segmentation	Relevant comorbidities	Summary of findings
[38] CMR	101/30	Yes	CAD (0%) DM (17%)	LGE associated with reduced hyperaemic MBF^M^
[29] CMR	100/0	Yes	CAD (5%) DM (10%)	LGE transmurally dissociated from hyperaemic MBF impairment^U^
[97] CMR	35/14	Yes	CAD (N/A) DM (0%)	LGE associated with reduced hyperaemic MBF^U^
[31] CMR	35/0	Yes	CAD (0%) DM (0%)	LGE associated with reduced MPRI, rest and hyperaemic MBF^U+M−^
[106] CMR	20/10	Yes	CAD (0%) DM (N/A)	LGE associated with reduced MPR, hyperaemic MBF^M^. unclear if statistically significant
[41] CMR	105/0	No	CAD (0%) DM (11%)	ECV, not LGE, associated with MPRI < 2^M^ LGE associated with reduced MPRI^U^
[101] CMR	75/0	No	CAD (N/A) DM (N/A)	LGE, ECV, T1 and T2 associated with stress perfusion defects^M^
[120] CMR	55/0	No	CAD (0%) DM (N/A)	ECV, LGE, T1 associated with hypoxia^U^
[100] CMR	47/21	No	CAD (0%) DM (0%)	LGE associated with increased time to perfusion peak^U^
[116] PET	34/0	No	CAD (N/A) DM (N/A)	LGE associated with reduced hyperaemic MBF, particularly if visually transmural LGE^U^
[46] CMR	37/31	No	CAD (N/A) DM (N/A)	LGE associated with reduced MPRI^U^
[99] CMR	22/13	No	CAD (N/A) DM (N/A)	LGE associated with lower maximum perfusion upslopes^M^
[33] CMR	12/9	No	CAD (N/A) DM (N/A) All < 30 years	LGE associated with lower stress/rest ratio of maximum perfusion upslopes^M^
[28] PET	47/0	N/A	CAD (0%) DM (11%)	Delayed enhancement visually coincided with stress perfusion defects^U^
[121] Scintigraphy	6/0	N/A	CAD (0%) DM (N/A)	LGE visually coincided with stress perfusion defects^U^

Epi-endo segmentation refers to whether imaged LV segments were further divided into epicardial and endocardial subsegments. ^U^Association does not control for wall thickness; ^M^Association controls for wall thickness; ^U+M−^Association is lost when controlling for wall thickness

*CAD* coronary artery disease, *ECV* extracellular volume, *DM* diabetes mellitus, *MPRI* myocardial perfusion reserve index, *CMR* cardiovascular magnetic resonance, *PET* positron emission tomography, *N* number of HCM patients, *n* number of non-HCM controls, *N/A* not assessed.

Imaging studies that did not perform epi-endocardial segmentation, but controlled for wall thickness, found independent associations between perfusion impairment and either LGE or extracellular volume [33, 41, 99, 101]. One study with epi-endocardial segmentation and control for wall thickness reported an independent association between LGE and hyperaemic MBF [38], but two smaller similar studies found that either this association was lost after controlling for wall thickness [31], or the differences in perfusion with/without LGE were modest [106]. Patient-wise analyses typically found ischaemia-fibrosis associations [10, 28, 29, 32, 36, 42, 102, 117, 118], with reported exceptions [40, 73].

Collectively, these findings suggest colocalization of pathology (microvascular dysfunction and fibrosis) and the potential for ischaemia to promote the fibrosis phenotype. However, it is possible that imaging difficulties in discerning regions of fibrosis and hypoperfusion could overestimate the fibrosis-ischaemia association [36]. Furthermore, assessment of LGE alone can miss any association between interstitial fibrosis and small vessel disease [59] and in this regard, it is worth considering the study in which ischaema was independently associated with extracellular volume, but not LGE [41].

Extracellular matrix expansion in myocardial regions remote from microvascular dysfunction has been suggested to be triggered by pro-hypertrophic transforming growth factor beta (TGF-β) signalling secondary to the sarcomeric mutation [119]. This non-ischaemic aetiology of fibrosis would support the findings of other studies where fibrosis can be seen in hearts with normal perfusion [28, 39].

## Clinical manifestations of myocardial ischaemia in HCM

The main clinical manifestations of myocardial ischaemia in HCM patients are angina and dyspnoea [17, 25, 122, 123], alongside dynamic changes on exercise/vasodilator-induced stress electrocardiogram (ECG) testing [20, 124].

Perfusion abnormalities may also be present in the absence of symptoms [14, 20, 22, 23, 40, 41, 102], so the relationship between pathology and symptoms may not always be consistent. Of interest, post-mortem studies of HCM patient hearts have noted a relative absence of historical symptoms among individuals with transmural infarction [53]. This confirms the potential for ischaemia to be silent, possibly through small fibre neuropathy reducing afferent pain signal detection. Mildly abnormal troponin levels are also common among HCM patients, reported in 74% [125], and likely represent myocyte injury or necrosis thought to be exacerbated by myocardial ischaemia.

## Mechanisms of myocardial ischaemia progression in HCM

**Fig. 2:**
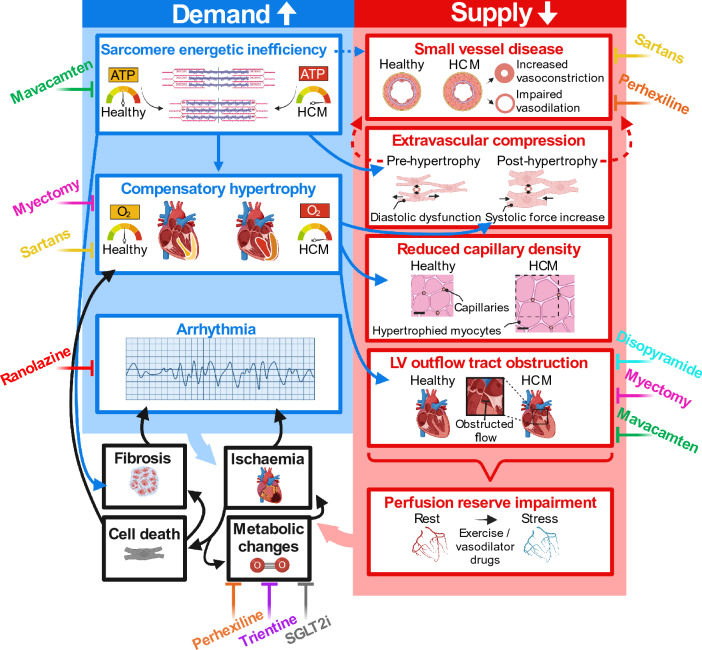
Mechanisms involved in progression of ischaemia in HCM. Disease factors in HCM constrain myocardial blood supply and increase energetic demands. Likely causal (solid arrows) and possibly causal (dashed arrows) relationships between pro-ischaemic disease factors are denoted, alongside potential anti-ischaemic therapies [76]

Figure 2 summarises the likely progression of factors affecting oxygen supply and demand in HCM. Early in development, ATP depletion arising from the sarcomeric mutation likely causes stress oxygenation impairment [46, 89], and could contribute to phenotype development through SERCA ATP starvation, calcium accumulation and hypertrophy via calcium signalling [126]. The cause of subsequent metabolic abnormalities in HCM is unclear [91, 127, 128]. Small vessel disease, present even in those < 1 year of age [51], could emerge during embryonic development driven by sarcomeric mutations [129], and is proposed to be one of the earliest factors in the cascade of events related to ischaemia [3]. Pre-hypertrophic diastolic dysfunction secondary to the sarcomeric mutation [74, 75] could contribute by limiting the time for myocardial relaxation, leading to increased rest MBF [76], which could promote vascular remodelling through increased shear stress. However, perfusion impairment reported in the absence of diastolic dysfunction [98] suggests that diastolic dysfunction is not the only contributor. Early perfusion defects seen on stress imaging of HCM patients may occur due to the microscopic steal phenomenon secondary to small vessel disease, or due to an abnormal vasomotor response of diseased myocardium to pharmacologically induced vasodilation [38].

As cells become hypertrophied, either to compensate for cells lost through ischaemia-induced fibrosis or other pathological processes, ischaemia is further promoted by local reductions in small vessel density [61], greater energetic demands, increased extravascular compression, and LVOT obstruction. Post-hypertrophy diastolic dysfunction (possibly exacerbated by ionic remodelling in hypertrophied segments [130]) may be a key contributor to ischaemia as the diastole-specific perfusion reserve is more strongly correlated with wall thickness than the time-averaged perfusion reserve [48]. Moreover, the increased oxygen demand of hypertrophy leads to resting vasodilation, giving rise to ‘maxed out’ vasodilation at rest [31, 43, 69, 71, 131] and explains why perfusion defects are more prevalent in cohorts with hypertrophy [42] than those without [43].

With disease progression, the ischaemic threshold is incrementally lowered, such that acute episodes of ischaemia are inducible despite non-stenotic epicardial coronary arteries. In some HCM patients, transient increases in energetic demands, such as during AF-induced increases in ventricular pacing [11] or during exercise [132], are sufficient to trigger an ischaemic episode, which can precipitate lethal ventricular arrhythmias.

In patients with the most severe small vessel disease and insufficient capillary density, ischaemia leads to gross macroscopic transmural scarring [3, 12, 59, 62, 111], which contributes amongst other factors (mitral regurgitation [42]) to the 2–16% subset of HCM patients that progress to end-stage disease [133, 134]. Myocyte death eventually leads to total replacement of myocardial regions with fibrosis, such that affected regions are devoid of myocytes that could hypertrophy, leading to wall thinning, LV stiffening and systolic dysfunction [39, 42, 135]. In this stage, LVOT gradients are resolved at the peril of reduced ejection fraction.

## Prognostic value of myocardial ischaemia in HCM

### Composite endpoints

The studies in Table 3 have analysed the association of ischaemia in HCM with adverse events using composite endpoints, where non-arrhythmic events such as heart failure or all-cause death were included. Although many studies found hypoperfusion to be associated with adverse outcomes on multivariate analysis [4, 25, 35, 121, 124, 136], they did not assess focal fibrosis burden, which is an independent predictor of mortality in HCM [137]. In the study that did account for fibrosis confounding, exercise WMAs were an independent predictor of cardiac events—not visual assessment of perfusion defects on CMR [32]. Although WMAs are not specific for myocardial ischaemia, a strong association between perfusion defects and WMAs has been reported [32]. In the largest study to date, exercise WMAs were substantiated as a possible risk factor in HCM, particularly when considered alongside non-invasive quantitative perfusion reserve measurements [35].

**Table 3:** Studies that investigated the association between ischaemia and prognosis in HCM patients, where composite endpoints were used

References Year	N/n	Ischaemia measure	Follow-up (years)	Summary of findings
[141] 1996	62/0	^123^I-BMIPP SPECT metabolic impairment score ≥ 30 vs. < 30	3 ± 1	11 × risk of death^U^
[124] 1997	79/0	ST segment depression on dipyridamole ECG	6 ± 1	6 × risk of cardiac events^U^ Independent predictor of cardiac events^M*^
[22] 1998	216/0	Fixed perfusion defect(s) on SPECT	3 ± 2	Unrelated to HCM-related death^M*^ 2 × risk of historical syncope / ventricular fibrillation^U^
[4] 2003	51/12	Lowest tertile of hyperaemic MBF on PET	8 ± 2	10 × risk of cardiac death^M*^ 20 × risk of unfavourable outcome^M*^
[23] 2004	101/0	Fixed perfusion defect(s) on SPECT	6 ± 3	Unrelated to cardiac death^U^ 3–4 × risk of severe complications^U^
[25] 2008	68/74	Perfusion reserve < 2 on echo	2 ± 1	4 × risk of cardiac events^M*^
[121] 2011	55/0	Anterior junction stress score > 2 on scintigraphy	6	8 × risk of cardiac events^M*^
[142] 2013	35/0	LAD perfusion reserve < 2 on Doppler catheter	9	3–6 × risk of cardiac events^U^
[32] 2014	148/0	Perfusion defects and exercise WMAs on CMR + echo	7 ± 2	Perfusion defects associated with 5 × risk of cardiac events^U+M−^ Exercise WMAs associated with 400 × risk of cardiac events^M^
[35] 2016	706/0	Perfusion reserve < 2 and WMAs on echo	4 [2, 6]	5 × risk of cardiac events^M*^
[136] 2016	100/0	Lowest tertile of hyperaemic MBF on PET	4 ± 2	7 × risk of unfavourable outcome^M*^
[10] 2021	91/0	Perfusion defects on SPECT	8 [4, 11]	3 × risk of cardiac events^U^

Follow-up durations given as mean or median, with variability given as ± standard deviation or [lower quartile, upper quartile], where available. ^U^Univariate analysis; ^M^Multivariate analysis controlling for fibrosis; ^M*^Multivariate analysis not controlling for fibrosis; ^U+M−^Association is lost when controlling for fibrosis

*CMR* cardiovascular magnetic resonance, *PET* positron emission tomography, *SPECT* single-photon emission computed tomography, *WMA* wall motion abnormality, *N* number of HCM patients, *n* number of non-HCM controls

Overall, the studies in Table 3 demonstrate that quantitative assessment of ischaemia identifies a subgroup of HCM patients at high risk of adverse outcomes, with the hyperaemic MBF threshold on PET optimally associated with outcomes estimated as 1.1–1.35 ml/min/g [4, 134, 136]. There is mixed evidence from studies of visual perfusion assessment, suggesting that further studies may benefit from quantitative perfusion analysis.

The prognostic value of other pathophysiological mechanisms, such as myocardial bridging, is debatable. Although bridging has been shown to predict poorer prognosis in one paediatric cohort [138], this association was not consistent elsewhere [139]. Furthermore, Sorajja et al. did not observe an association between bridging and poor outcomes in adult HCM patients [140].

### Arrhythmia and sudden cardiac death

Acute myocardial ischaemia (in the absence of epicardial coronary stenoses) may be an important cause of fatal arrhythmias and SCD in HCM [5–7, 143], as multiple case reports describe arrhythmias precipitated by acute myocardial ischaemia in young patients [11, 132, 144]. In a postmortem study of 19 young (≤ 35 years) SCD victims with HCM, 11 had physical evidence of acute-subacute myocardial ischaemia (coagulative necrosis, neutrophilic infiltrate, myocytolisis, granulation tissue healing, infarction) in the septal myocardium [47]. Multiple foci of transmural infarction have also been reported in some deceased HCM patients, despite having normal epicardial coronary arteries [53].

Numerous studies (Table 4) have analysed the association between ischaemia and arrhythmia in HCM. Many studies accounted for confounding by fibrosis and found independent associations between ischaemia measurements and arrhythmia [34, 40, 44, 109]. The specific association was variable across studies, and it is unclear the extent to which differences in imaging modality, protocol and cohort may have contributed to this variety. Importantly, however, there is some suggestion that measurements of MBF heterogeneity predict arrhythmic risk [109, 145], motivating further study of regional perfusion quantification. There is further evidence from echocardiographic and scintigraphic studies that ischaemia is associated with syncopal episodes in HCM [15, 18].

**Table 4:** Studies that investigated the association between ischaemia and arrhythmia in HCM patients

References Year	N/n	Ages (years)	Ischaemia measure	Summary of findings
[146] 1997	84/0	43 ± 12	Perfusion reserve / transmural MBF gradients on PET	Unrelated to syncope or NSVT on Holter^U^
[147] 2009	95/0	41 ± 15	Lower hyperaemic MBF on PET	Associated with history of AF^M*^
[102] 2011	62/35	47 ± 16	Lower rest MBF on CMR	Associated with NSVT on Holter^U^
[34] 2015	80/0	50 ± 18	Rest perfusion abnormalities on CMR	Associated with NSVT on Holter^M^
[109] 2018	133/0	50 ± 15	High hyperaemic MBF heterogeneity on PET	4 × risk of VT on ICD electrogram/Holter^M^
[7] 2019	104/0	65	Decreased perfusion on angiography	Associated with paroxysmal supra-VT and VT on Holter^U^
[145] 2020	25/0	57 ± 13	Decreased hyperaemic endo/epicardial MBF ratio on PET	Associated with NSVT on ICD electrogram^U^
[40] 2020	115/0	52 ± 11	Perfusion defect on CMR	6 × risk of NSVT on Holter^M^
[10] 2021	91/0	14 [10, 16]	Perfusion defect on SPECT	Associated with NSVT on follow-up^U^
[148] 2021	32/0	62 ± 16	Reduced perfusion upslope on CMR	Associated with VT on Holter^U^
[44] 2021	75/0	55 ± 15	Perfusion defect on CMR	2 × risk of supra-VT on Holter, but not VT^M^

Ages given as mean or median, with variability given as ± standard deviation or [lower quartile, upper quartile], where available. ^U^Univariate analysis; ^M^Multivariate analysis controlling for fibrosis; ^M*^Multivariate analysis not controlling for fibrosis

*CMR* cardiovascular magnetic resonance, *ICD* implantable cardioverter defibrillator, *(NS)VT* (non-sustained) ventricular tachycardia, *AF* atrial fibrillation, *PET* positron emission tomography, *SPECT* single-photon emission computed tomography, *N* number of HCM patients, *n* number of non-HCM controls

### Heart failure

Some studies report an association between ischaemia and heart failure. Hamada et al. used Th-201 scintigraphy to study 48 HCM patients and found that development of heart failure was associated with perfusion impairment [107]. Similarly, Olivotto et al. used stress PET in 51 HCM patients and found that adverse LV remodelling and systolic dysfunction were predicted by quantitative assessment of perfusion [134]. SPECT imaging of 65 HCM patients also found an association between metabolic impairment and heart failure [141]. Similarly, perfusion and LGE CMR of 62 HCM patients found that reduced MBF was the only independent predictor of functional status when LGE and hypertrophy were accounted for [102]. However, in the largest longitudinal perfusion CMR study to date of 449 HCM patients, perfusion defects on visual assessment were unable to predict heart failure [42]. LGE progression may instead be a more prominent factor in the development of heart failure as an independent association has been reported [39].

### Confounding by coexisting pathology

In evaluating the independent prognostic contributions of perfusion defects and presence of LGE, not only are perfusion defects confounded by presence of LGE due to the possibly causal link between ischaemia and fibrosis in HCM [39], but some perfusion defects correspond to sites of LGE [40]. It is therefore unclear whether SCDs in HCM are caused by (i) primary ventricular arrhythmia related to fibrosis [137], or (ii) secondary ventricular arrhythmia during ischaemia [34]. Simultaneous echo and perfusion CMR in 148 HCM patients showed that cardiac event rates were highest when both LGE and exercise WMAs were observed [32], hence both factors may contribute to increased risk. This is further supported by the finding that both fixed and reversible perfusion defects have prognostic value [10], as perfusion abnormalities present at rest are thought to represent severe fibrosis [34]. Ischaemia may be relevant to the cases of juvenile SCDs in which replacement fibrosis is absent [12].

As a possible cause of ischaemia, LVOT obstruction is already recognised as a risk factor for SCD in adults [149] and children [150]. Further confounding may arise from atrial fibrillation due to its association with ischaemia [147].

### Exercise

Myocardial ischaemia in HCM is relevant in the context of evolving clinical guidelines on exercise restrictions. If SCD is related to exercise [151], then ischaemia is a plausible arrhythmic substrate through exercise-induced ischaemia [152], latent LVOT obstruction [66] and reduced diastolic filling time [77] during increased workload. Measurements derived from stress perfusion imaging could contribute to decisions made with patients on exercise, although at present there is limited clinical evidence that this would be useful.

Of note, two meta-analyses have shown that SCDs in young HCM patients are 75% more common in athletes than non-athletes [153, 154]. Age appears to be a factor in the association between exercise and SCD in HCM [155], with Weissler-Snir et al. reporting 80% of HCM-related SCDs in those ≤ 20 years old being related to exercise, compared to < 5% of those above 20 [151]. Interestingly, a study of 1380 HCM patients showed that, on multivariate analysis, only NSVT induced by exercise was associated with SCD—not NSVT generally [156]. In this analysis, 21% of patients with exercise-induced NSVT had preceding ST depression, and most were < 40 years of age. There are other varying reports of SCD-predictive measures derived from exercise [152, 157, 158], including the ventilation-to-CO_2_ (VE/ CO_2_) slope and anaerobic threshold during exercise, all of which could indicate prospensity for underlying myocardial ischaemia on cardiopulmonary exercise testing.

## Clinical perspective: myocardial ischaemia in HCM as a therapeutic target

The potential for ischaemia as an early therapeutic target in HCM [43] is reinforced by the improvements in myocardial perfusion and patient symptoms that typically accompany invasive surgical relief of LVOT obstruction [159, 160]. By reducing the perfusion sink, relief of LVOT obstruction may cause less vasodilatory reserve to be exhausted at rest, in addition to reductions in wall stress and extravascular compression.

Multiple promising studies of pharmacologic treatments with the potential to minimise ischaemic burden in HCM are ongoing or have been completed (Fig. 2). These include angiotensin receptor blockers, vasodilators, metabolic modulators, late sodium blockers, negative inotropes as well as novel allosteric myosin inhibitors.

Angiotensin receptor blockers (‘-sartans’), which target both vascular function and the TGF-β signalling pathway (also associated with the emergence of fibrosis [119]), have shown efficacy in limiting phenotype development. Although it is unknown whether sartans affect perfusion defects in HCM [43], candesartan and valsartan may attenuate the HCM phenotype [161–163], with valsartan being more efficacious in those with less hypertrophic remodelling [163].

Perhexiline, a vasodilator and metabolic modulator, recently showed lack of efficacy to improve exercise capacity in HCM patients with moderate to severe heart failure (trial NCT02862600). However, the findings of this interventional study may have been significantly influenced by the advanced disease progression into heart failure of the recruited patients and the choice of primary endpoint. RESOLVE-HCM (trial NCT04426578) is another study which is assessing the impact of perhexiline on LV hypertrophy [164]. This trial includes changes in oxygen-sensitive CMR measures as a secondary endpoint, which could directly evidence anti-ischaemic pharmacologic treatment. Trientine, a modulator of copper metabolism, is also being investigated in HCM (trial ISRCTN57145331). The novel class of metabolic modulator drugs, sodium-glucose co-transporter 2 inhibitors, may also have potential in HCM (trial NCT05182658). As a further potentially novel therapeutic target due to their effects on vascular function and metabolism [165], ceramides have been implicated in the development of various cardiovascular diseases [166], which may be relevant to HCM.

Late sodium blockers, which target the pathologically increased late sodium current in HCM cardiomyocytes [130], were hypothesised to ameliorate diastolic dysfunction [167] and thus downstream ischaemic effects. However, in RESTYLE-HCM (trial 2011-004507-20), ranolazine showed no efficacy in reducing diastolic dysfunction or pro B-type natriuretic peptide in non-obstructive HCM patients, despite finding a possible antiarrhythmic effect [168]. Potential amelioration of ischaemia-induced arrhythmia by ranolazine is also described in a recent study [169]. LIBERTY-HCM (trial NCT02291237) was terminated early due to a lack of efficacy of eleclazine administration [170]. Finally, although disopyramide ameliorates symptoms and reduces LVOT obstruction in HCM, these effects are attributed to its negative inotropic action rather than late sodium block [171].

Perhaps most promising is the novel allosteric myosin inhibitor mavacamten, which (in contrast to the drugs previously introduced) specifically targets the underlying pathogenic drivers of contractile dysfunction in HCM at the sarcomeric level [172]. Both the results of EXPLORER-HCM (trial NCT03470545) [173] and additional studies [174] have proven mavacamten effective at improving cardiac function in HCM patients, including the reduction of LVOT obstruction gradients. The marked reductions in N-terminal pro B-type natriuretic peptide and cardiac troponin I during mavacamten treatment indicate that the drug may reduce the extent of ischaemic injury in HCM [175], likely through attenuation of the downstream pro-ischaemic effects of sarcomeric impairment, as shown in Fig. 2.

## Future research

All the above routes towards a refined diagnosis and targeted treatment of myocardial ischaemia in HCM constitute important and promising prospects for future research into the amelioration of symptoms and risk of SCD in HCM. If successful, their integration into HCM risk stratification models and clinical guidelines is expected to yield significant advances for the management of this high-risk group of patients, as well as further insights on the overall contribution of ischaemia in cardiovascular disease. However, further research is needed to elucidate the clinical significance of ischaemia on imaging, and the relative contributions of the various pro-ischaemic mechanisms in HCM.

Although ischaemic HCM phenotypes are consistently identified as a high-risk subgroup on long term follow-up (Table 3), the role of imaging in assessing ischaemic burden for risk prediction needs rigorous testing. Reported ischaemia-arrhythmia associations are heterogeneous (Table 4) and might be explained by monitoring of ECG (Holter monitors) during conditions of rest to assess patients’ arrhythmic burden. Future work might consider whether ischaemia on non-invasive imaging is associated with arrhythmias on stress testing, particularly as some arrhythmias are preceded by ischaemic ECG changes [156]. Such multimodal approaches have already been evaluated in HCM, such as the combined use of perfusion CMR and echocardiography [32], demonstrating its potential to improve diagnosis and prognostic stratification of HCM patients.

Another consideration is that despite the strong age-dependence of SCD risk in HCM, most analyses relating ischaemia and arrhythmic risk were performed in midlife cohorts (Table 4), which may be more likely to have a fibrotic substrate due to the presence of more advanced structural LV remodelling. Future analyses of imaging for ischaemia in younger HCM cohorts may have distinct implications, given that exercise-induced ECG changes are emerging as predictive of outcomes [176].

Importantly, multi-centre studies to evaluate ischaemic burden are needed to elucidate the incremental value of perfusion imaging for ischaemic myocardial substrates over potentially irreversible substrates like LGE, particularly in subgroups where guidelines are less certain (ESC SCD risk < 6%).

## Conclusion

In this review, we have presented a comprehensive discussion of the latest evidence corroborating profound links between myocardial ischaemia, disease severity and prognosis. This notably broadens former studies by covering HCM-specific ischaemic factors, the impairment of perfusion by myocardial hypertrophy, the characteristic distributions of ischaemic burden in HCM ventricles, the relationship between (hypo)perfusion and fibrosis, mechanisms of ischaemia progression in HCM, its clinical manifestations and prognostic value. Altogether, our analysis substantiates myocardial ischaemia as a strong and multifactorial contributor to adverse LV remodelling, arrhythmia, and SCD events in HCM. Despite the strong associations reported, further studies are needed to understand which non-invasive methods of ischaemia assessment have independent prognostic value in HCM, over and above co-existing myocardial fibrosis and LVOT obstruction.
